# Colony Binary Classification Based on Persistent Homology Feature Extraction and Improved EfficientNet

**DOI:** 10.3390/bioengineering12060625

**Published:** 2025-06-09

**Authors:** Zumin Wang, Ke Yang, Jie Tang, Jun Gao, Yuhao Zhang, Wei Xu, Chun-Ming Huang

**Affiliations:** 1 School of Information Engineering, Dalian University, Dalian 116622, China; wangzumin@dlu.edu.cn (Z.W.); gaojun@s.dlu.edu.cn (J.G.); 2 Medical College, Dalian University, Dalian 116622, China; tangjiedlu@163.com; 3 School of Computer Science, Dalian University of Technology, Dalian 116024, China; zhangyuhao@mail.dlut.edu.cn; 4 Centre for Artificial Intelligence Driven Drug Discovery, Macao Polytechnic University, Macao SAR, China; xuwei_chn@foxmail.com

**Keywords:** colony, persistent homology, EfficientNet, image classification

## Abstract

Classifying newly formed colonies is instrumental in uncovering sources of infection and enabling precision medicine, holding significant clinical value. However, due to the ambiguous features of early-stage colony images in culture dishes, conventional computer vision (CV) classification algorithms are often ineffective. To achieve accurate and efficient colony classification, this paper proposes a high-precision method based on Persistent Homology (PH) and an improved EfficientNet. Specifically, (1) a PH feature extraction algorithm is applied to Candida albicans (CA) and Staphylococcus epidermidis (SE) colonies cultured for 18 h in Petri dishes to capture their topological information. (2) The Mobile Inverted Bottleneck Convolution (MBConv) module in EfficientNet is modified, enhancing the attention mechanism to better handle local small targets. (3) A novel self-attention mechanism named the Spatial and Contextual Transformer (SCoT), which is introduced to process information at multiple scales, increasing the resolution in orthogonal directions of the image and the aggregation capability of feature maps. The proposed approach achieves a high accuracy of 98.64%, a 10.29% improvement over the original classification model. The research findings indicate that this method can effectively classify colonies with high efficiency.

## 1. Introduction

Bacteria and fungi are both unicellular microorganisms, typically characterized by their minute size, rendering them invisible to the naked eye [1]. These microorganisms exhibit remarkable ecological adaptability and are widely distributed across various sites within the human body and numerous environments encountered in daily life [2]. Candida Albicans (CA) and Staphylococcus Epidermidis (SE) are, respectively, the most commonly isolated fungal and bacterial pathogens associated with bloodstream infections globally [3,4]. These two microorganisms can coexist or exist independently within the human body, potentially causing a variety of infectious diseases. Compared to single-species cultures, the coexistence of CA and SE significantly increases biofilm density and is accompanied by enhanced drug resistance [5,6]. Therefore, accurately determining infection status and distinguishing the types of infectious bacteria are particularly important for the diagnosis and treatment of diseases.

The principle of bacterial culture is to inoculate the microbial sample onto a suitable medium and to provide suitable growth conditions so that it can grow and reproduce [7]. The 16sRNA bacterial identification is a common and cost-effective method for identifying bacterial RNA sequences. Then, the technique for the identification and analysis of the bacteria is by comparing with the database [8]. Many 16sRNA-based sequencing technologies (such as the Illumina platform) can only generate shorter sequences (<500 bp), which limits the coverage of the entire 16sRNA gene [9]. During the PCR amplification process, there may be a primer bias, making the amplification efficiency of 16sRNA genes of some microorganisms higher, thereby leading to distortion of the community composition [10]. The identification of bacteria by 16sRNA requires cumbersome steps and takes a lot of manpower and time. Therefore, there is an urgent need to develop a precise and rapid method for classifying colonies that requires minimal medical knowledge and reduces human intervention, in order to assist doctors in making diagnoses.

With the rapid development of computer vision technology, it has become possible to use various algorithms to extract image features and build classification models to solve medical image classification problems. In addition, the emergence of novel technologies and their integration with medical systems have enabled the early detection of diseases, providing new opportunities in the field of medical image classification [11]. The combination of image processing technology and various classifiers is often used as an effective means to identify laboratory image samples [12]. In the field of computer vision, feature extraction algorithms play a crucial role. These technologies use diverse mathematical and statistical methods to extract or filter features, effectively preserving essential information while eliminating noise or irrelevant variables, thus significantly improving the performance and classification capabilities of the model. Meanwhile, traditional classification methods analyze data features based on mathematical and statistical principles, constructing models that assign data points to predefined categories, thereby achieving the accurate prediction and classification of unknown data. With ongoing technological advancements, deep learning models have attracted widespread attention and have led to significant breakthroughs in image classification tasks. These models can automatically learn and extract hierarchical features from images, making them particularly well suited for processing complex medical images. The continued progress in these technologies presents new opportunities in medical image classification, promising a faster and more accurate classification of colony image data and thus assisting medical professionals in clinical diagnosis.

This study proposes a novel colony classification algorithm that integrates the advantages of the PH algorithm and EfficientNet [13]. The EfficientNet model is optimized, including improvements to its MBConv module and the innovative introduction of the SCoT self-attention mechanism, to enhance its performance in colony image classification. Particularly, in the early stage of colony cultivation, when image features are indistinct, the proposed method achieves accurate recognition and classification of small target colonies. The aim is to reduce manual intervention, shorten the diagnostic cycle, and assist doctors in rapid and accurate diagnosis and treatment. The main contributions of the proposed method are as follows:

1. The experimental dataset selects colonies that have grown in a Petri dish for 18 h for identification, eliminating the need to wait for significant colony features to appear or to use sequencing instruments. This approach establishes a foundation for improving the overall identification speed of colonies, reducing the cost of manual judgment, and decreasing the dependence on medical expertise in the identification process.
2. This study fully leverages the ease of integration and the advantage of processing deep features of the PH algorithm, successfully extracting the topological features of CA and SE. It effectively addresses the challenge of vague and difficult-to-distinguish features in the early stage of colony culture, significantly enhancing the classification accuracy of the model when dealing with medical images with indistinct characteristics. Furthermore, this research offers robust support for the in-depth exploration of the structure and characteristics of colony growth.
3. This study utilizes the efficiency, computational lightweightness, and advantages in sensitivity and specificity of the EfficientNet model. It optimizes the MBConv module within EfficientNet by integrating the Efficient Channel Attention (ECA) mechanism, constructing the EMBConv architecture. This approach mitigates the negative effects caused by dimensionality reduction in the original module, reduces computational complexity, and enhances its performance in handling small local targets.
4. Prior to the tail convolution of the model, this study incorporates the SCoT self-attention mechanism, which comprehensively considers the contextual relationships and spatial channel information of the image. Through multi-scale processing, it enhances information integration, thereby improving the resolution of input image data in orthogonal directions and the aggregation capability of the feature map.
5. In this study, five evaluation metrics—accuracy, precision, recall, F-score, and Matthews Correlation Coefficient (MCC)—are introduced to comprehensively assess the model’s performance, significantly enhancing the generalization capability of the results.

This research not only enhances the accuracy of existing colony classification methods but also reduces the time and cost of manual judgment required during colony cultivation. This advancement is of great significance in promoting the further development of deep learning theory and technological innovation in the fields of microbiology and pathology, and it contributes to a comprehensive exploration of the structure and characteristics of colony growth.

## 2. Related Work

### 2.1. Feature Extraction Algorithms

In traditional feature extraction algorithms and their improvements, Nagwan [14] proposed a hybrid processing technology LR-PCA based on logistic regression (LR) and principal component analysis (PCA) for selecting important principal components to achieve further classification. However, it is not suitable for nonlinear structured data, and the dimensionality reduction approach of PCA inevitably causes certain feature loss. Joseph [15] applied 32 Gabor filters and Sobel edge detection to enhance features and built a dual-channel Gabor network based on attention for the accurate classification of anomalies. Gabor is effective at capturing image texture features, but its high computational cost and complex parameter representation limit its application. Y Peng et al. [16] proposed a Persistent-Homology-guided network (PHG-Net) based on the Persistent Homology algorithm for extracting structural features from convolutional neural networks (CNNs) or Transformer feature maps and fused these features with deep learning extracted feature maps. Persistent Homology can extract deep topological structure features, and compared with other feature extraction algorithms, Persistent Homology can retain the correlation and structural features within the data, rather than being limited to single linear features. It is also convenient to be used in combination with CNNs and other deep learning classification networks, but it similarly suffers from the problem of high computational complexity.

### 2.2. Classification Algorithms

#### 2.2.1. Traditional Classification Algorithm

In traditional classification algorithms, Yuzhu Li et al. [17] designed a bacterial colony forming unit (CFU) detection system based on a thin-film transistor (TFT) image sensor array, which enables the rapid detection and counting of colonies and identification of bacterial species. Ilya et al. [18] employed a subpixel correlation method to identify changes in continuous laser speckle images, thereby facilitating the visualization of specific areas within colonies that indicate microbial growth and achieving colony classification under non-white light illumination. V. Babenko [19] proposed a method for constructing a classifier within the random forest algorithm class based on genetic algorithms and the analytic hierarchy process, used for detecting medical image pathology, but inevitably carries the risk of overfitting. Shobhana et al. [20] divided breast thermal imaging medical thermography data into quadrant regions, introducing the support vector machine with a radial basis function kernel (SVM-RBF) classifier for the upper outer quadrant and the entire breast image, achieving an accuracy rate of 85.17%, but also resulting in significant memory consumption, which is unfavorable for further promotion and medical use. Although the aforementioned methods have demonstrated potential in extracting image features and have achieved colony classification to a certain extent, challenges remain in adaptively extracting discriminative features, resulting in limited robustness [21]. Additionally, these approaches typically require sophisticated experimental equipment, incur high costs, involve complex operations, and demand extensive microbiological expertise during experimentation, thereby restricting their broader adoption and application.

#### 2.2.2. Deep Learning Algorithm

As technology advances, deep learning classification networks are gradually replacing traditional classification algorithms, capturing the attention of scholars in the field of medical image classification. Pramudya et al. [22] evaluated four classification models for medical image classification, with EfficientNet-B0 and ResNet-50 outperforming other CNN models with classification accuracy rates of 85.12% and 87.59%, respectively. EfficientNet-B0 stands out in terms of parameter usage and computational resource efficiency, and its sensitivity and specificity are also impressive, which demonstrates the superiority of the EfficientNet network to some extent. Yunfeng Chen [23] constructed a classification model by combining Inception and ResNet neural networks and incorporated a self-attention mechanism for feature classification, effectively classifying lung infections. Jiawei Sun [24] proposed a thyroid nodule classification model TC-ViT that combines contrastive learning and Vision-Transformer (ViT), effectively capturing the overall features of thyroid nodules and improving the accuracy of diagnosis and the specificity of biopsy recommendations, but this method is less effective for small sample datasets. Abishek et al. [25] proposed an extended EfficientNet-B0 based on contour extraction (CE-EEN-B0) for identifying brain tumor MRI images, with experiments showing that the network can achieve an accuracy rate of 97.24% on limited datasets. A comprehensive comparative analysis indicates that the EfficientNet network is more suitable for classification problems involving small datasets and complex features, such as colony classification. While deep learning networks ensure robustness and certain classification advantages, their classification accuracy advantage is not significant when dealing with medical images with unclear feature information and blurred boundary information, and further optimization with variants such as self-attention mechanisms is needed.

## 3. Materials and Methods

### 3.1. Data Collection and Processing

**Figure 1:**
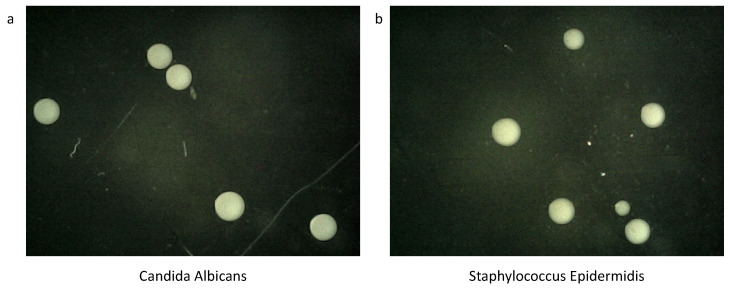
CA and SE data collection diagram. (**a**) represents the morphology of CA colonies after 18 h of cultivation in the medium, while (**b**) depicts the growth morphology of SE colonies under the same conditions. At this stage, the two types of colonies have essentially formed. Collecting the dataset now can significantly reduce the time required for colony differentiation.

In this paper, a self-collected dataset was used. Candida albicans and Staphylococcus epidermidis were inoculated in a TSB medium [26], cultured at 37 °C for 18 h, OD = 0.3, and the calculated bacteria amount was 2.4 × 10^8^. Then, TSB was used to dilute the original bacteria solution to 10-1, -2, -3, and -4, respectively. To the power of negative 5, 50 μL was applied in the TSA solid medium for flat cotton swab coating, cultured in a 37 °C temperature box for 18 h, and colony data were obtained as shown in Figure 1.

At the conclusion of the 18-h incubation period, the solid culture medium predominantly exhibits white, circular, and variably sized colonies. These characteristics facilitate the rapid identification and classification of microbial samples in the diagnostic process of microbiology. Capturing images of the colonies at this stage can substantially reduce the time required for colony differentiation and expedite the diagnostic process. In this study, an S4T digital microscope was utilized to capture images at a resolution of 640 × 480, followed by the selection and trimming of the photographed colony images.

**Figure 2:**
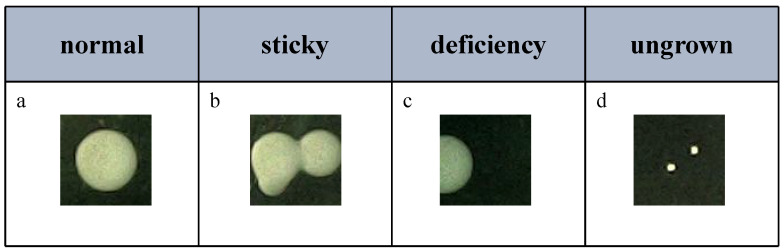
Screening images. Figure (**a**) shows normally collected colony data, Figure (**b**) depicts colonies that are adhered to each other, Figure (**c**) illustrates colony images with missing or truncated parts, and Figure (**d**) represents colonies that have not reached maturity. After screening, Figure (**a**) is retained, while Figures (**b**–**d**) are excluded.

Screening: A stringent screening procedure was implemented for the collected colony dataset to exclude colonies that did not meet the quality standards, as illustrated in Figure 2. Specifically, colonies that were adhered, partially captured, or incompletely grown were removed. This screening process ensured the integrity and uniformity of the retained colony dataset, thereby providing a high-quality data foundation for subsequent analysis and classification studies.

**Figure 3:**
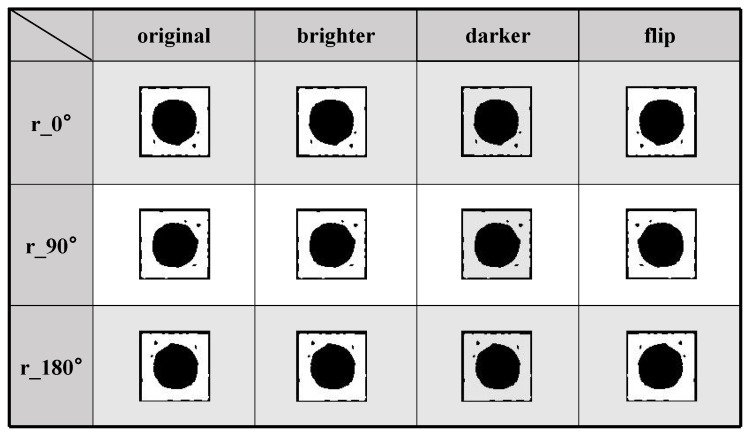
Data enhancement processing. The PH feature marking map of the augmented dataset after data augmentation, where this data augmentation method does not affect the extraction of topological features from the PH image.

Cropping: To ensure consistency and reliability in the input data for the colony identification and classification model, precise cropping was performed on the screened colony images. This process aims to maximize the retention of valid data without introducing invalid data, which facilitates subsequent image processing and analysis steps. As shown in Figure 3, the dataset after screening and tailoring was expanded by the data enhancement method, and the final dataset contained 3168 CA and 3096 SE. Datasets are classified as training sets and test sets in an 8:2 fashion.

### 3.2. Framework

This paper proposes a research approach for the binary classification of bacterial colonies, conducting a discriminatory study on CA and SE, which have the same pathogenic effects and exhibit insignificant differences in their initial growth. The aim is to reduce the required time for colony cultivation, lower the dependence on professional expertise, reduce the required time for colony cultivation, lower the dependence on professional expertise, and enhance the classification accuracy.

**Figure 4:**
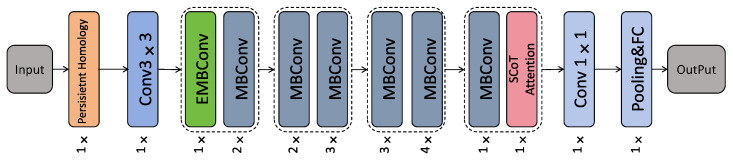
Model the overall network architecture. The architecture comprises a PH feature extraction module, a standard convolution module with a 3 × 3 kernel size, a combined module of EMBConv and MBConv, two self-combined modules of MBConv, a combined module of MBConv and SCoT self-attention, a convolution module with a 1 × 1 kernel size, and an average pooling with a fully connected output module.

This study consists of feature extraction and classification processing. In the feature extraction stage, the key feature regions of the images are enhanced using Persistent Homology (PH) on the expanded dataset. In the classification processing module, the SCoT_EfficientNet classification model is constructed by improving the EfficientNet-B0 model [13]. The MBConv module in EfficientNet is redesigned based on the convolutional network design idea, and the Efficient Channel Attention (ECA) convolutional attention [27] is introduced after the first convolution to construct the EMBConv structure. The SCoT self-attention mechanism is innovatively added before the final convolution to further refine feature representations, and the linear classifier Softmax is used to classify the features and output the recognition results. The specific method and internal structure are shown in Figure 4.

After being processed by the PH algorithm, the model can learn the deep topological features and potential structures of the colony data, thereby enhancing the model’s capability for accurate classification. This will provide the classification part of the model with higher-quality input features, allowing it to better learn and reconstruct the input data, thus more accurately classifying colony samples. By inputting the data into the SCoT_EfficientNet model for classification, the proposed method can achieve high computational efficiency and lightweight characteristics, while being able to accurately identify the features extracted by PH, further improving the classification accuracy. This integration strategy not only fully utilizes the integration-friendly characteristics of the PH algorithm and its advantage in processing deep features but also significantly improves the classification accuracy of the model when dealing with medical images with unclear features, effectively reducing computational costs.

### 3.3. Persistent Homology

Topological data analysis (TDA) is a technique that uses topological principles and methods to analyze data. As a new field, it considers the application of topology in data analysis [28,29], with the goal of identifying and understanding shapes, structures, and patterns in data and providing new insights. PH is a mathematical tool used in algebraic topology, a field of study that focuses on the shape and structure of data. Its core is to identify and quantify topological features such as connected components, voids, and holes in a dataset by constructing a series of topological spaces based on the data and analyzing how the homology groups of these spaces change as parameters (usually related to distance or scale) change. This allows for a method of capturing the essence of the data without dimensionality reduction, thereby providing a comprehensive understanding of the data as a whole [30].

In the early stages of colony growth, colonies typically have a clear circular or oval outline, with a uniform color and smooth edges [12]. Through the analysis of PH, we can quantify these morphological features such as the size, shape, and structural stability of the colony. The advantage of this method is that it can effectively handle minor noise and background unevenness in the image, providing a robust means of colony recognition and description. This allows us to identify and quantify topological features such as connected components, voids, and holes in the colony dataset, enabling a method of summarizing the full data without dimensionality reduction [31]. Additionally, since no complex parameter settings are required, PH provides an efficient tool for the rapid and accurate analysis of early colony images, helping us better understand the initial stages of microbial growth.

#### 3.3.1. Vietoris–Rips (VR) Complex

The VR complex algorithm is one of the standards for PH computation. The fundamental idea is to construct a series of simple geometric structures among the points in the given dataset, thereby understanding the topological properties of the data by observing the connection patterns of these shapes [32].

**Figure 5:**
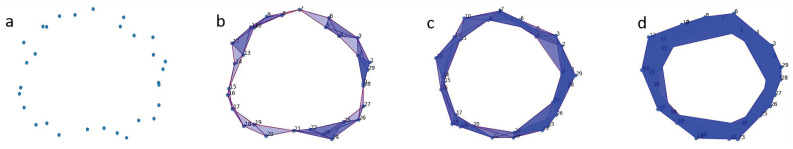
Topological feature extraction graph under different σ. (**a**) is the point cloud representation of image feature points. (**b**–**d**) are the topology images constructed when parameters σ are defined as 3, 4, and 6, respectively.

Specifically, the steps of the VR complex approach are as follows:

- Build a point cloud. A set *P* containing data points is generated from the colony data, which is the point cloud.
- Determine the parameters. Select a parameter σ that represents the radius of the build shape. σ determines the maximum distance between two points in *P* that can form a connection. As shown in Figure 5, topological feature extraction graphs formed by different parameters σ are different.
- Construct complex [33]. A dotted ball of radius σ is drawn around each point in P, and lines are drawn between this point and all other points in its circle, thus constructing a topological complex that best matches the characteristics of the colony.
  This part of the algorithm has two main steps:
  1. Construct a neighborhood plot of point set data. A domain graph is an undirected weighted graph (G,ω), where G=(V,E), *V* is the set of vertices, *E* is the set of edges, and weight ω:E→R is the mapping of each edge to the real numbers. Edges are obtained by linking examples defined by σ. Just like Formula (1):
    (1) $$E_{\sigma }=d(u,v)|d(u,v)\leq \sigma ,u\neq v\in V$$
    where d(u,v) is the distance function between two points. The weight function simply sets the weight of each edge to equal the distance between two points on the edge. Just like Formula (2):
    (2) $$\omega \left(u,v\right)=d\left(u,v\right),\forall u,v\in E_{\sigma }\left(V\right)$$
    Thus, the colony image is generated to form an undirected weighted neighborhood graph composed of a feature points set, which is used for the next calculation.
  2. In the first step, the generated field map forms the VR expansion. Combined with the results of the previous step, the given domain figure (G,ω) is obtained. The weight filtering of VR complex R(G) is given by Formula (3):
    (3) R(G)=V∪E∪τ|τ2⊆E
  For τ∈R(G):
    (4) $$\omega \left(\tau \right)=\left\{\begin{array}{c} \;0\;,\;\;\;\tau =v\in V\\ \omega (u,v),\;\;\tau =u,v\in E\\ \max_{\epsilon \subset \tau }\omega \left(\epsilon \right),\;\;otherwise \end{array}\right.$$
    In general, a ball around a point in *d*-dimensional space is a generalization of the ball around that point in (d−1) dimensional space (the ball refers to the set of all points in space that are the same distance from a point). So, the ball in *R* is a line segment around a point, the ball in $R^{2}$ is a circle, the ball in $R^{3}$ is a sphere, and so on, forming the spawn−VR complex. As shown in Formula (5), the complex structure is contained in proportion σ, and for all subsets τ of *P* in set $V_{\sigma }\left(P\right)$, the distance between all its different points is not greater than the parameter σ:
    (5) $$V_{\sigma }\left(P\right)=\left\{\tau \subseteq P|d\left(u,v\right)\leq \sigma ,\forall u\neq v\in \tau \right\}$$

- Analyze the topology. By analyzing the topology of the constructed complex, topological information about the colony dataset, such as connectivity and the presence of holes, can be obtained. First, the homology group of a simple complex is calculated. Considering simplex complex $V_{\sigma }\left(P\right)$ as a linear combination of integer bit coefficients λ, $\lambda_{1}\tau_{1}+\lambda_{2}\tau_{2}+\ldots +\lambda_{k}\tau_{k}$, one can define group addition to form a group:
  (6) $$\Sigma \lambda_{i}\left(u_{i},v_{i}\right)+\Sigma u_{i}\left(u_{i},v_{i}\right)=\Sigma \left(\lambda_{i}+u_{i}\right)\left(u_{i},v_{i}\right)$$
  Its identity element is 0, forming the Abelian group, that is, the chain group, and then the *d*-dimensional homology group of *K* of the simple complex is defined as:
  (7) $$H_{d}\left(K\right)=Z_{d}\left(K\right)/B_{d}\left(K\right)$$
  where $C_{d}\left(K\right)$ represents the *d*-chain group on the simplicial complex *K*, and its boundary homomorphism is mapped to $\delta :C_{d}\left(K\right)\to C_{d-1}\left(K\right)$, and the homomorphism kernel of the identity element obtained through the submapping is a subgroup of the *d*-dimensional chain group $C_{d}\left(K\right)$, and is also a *d*-dimensional closed chain group, denoated as $Z_{d}\left(K\right)$. All edges in $C_{d}\left(K\right)$, that is, the homomorphic image obtained by homomorphic mapping $\delta :C_{d+1}\left(K\right)\to C_{d}\left(K\right)$, are subgroups of the *d*-dimensional chain group $C_{d}\left(K\right)$, and also subgroups of the D-dimensional closed group chain $Z_{d}\left(K\right)$, referred to as the *d*-dimensional edge group $B_{d}\left(K\right)$, where $B_{n}\leq Z_{n}\leq C_{n}$. The connectivity number of the simple complex is obtained from this calculation. The columns of the matrix are regarded as a set of basis vectors: $\beta_{1},\beta_{2},\ldots ,\beta_{k}$, then the dimension of the space composed of these column vectors is the rank of the matrix, and the connected number $b_{n}=rank\left(Z_{n}\right)-rank\left(B_{n}\right)$ is defined to obtain the topology information of the complex.

#### 3.3.2. Filtration

The filter flow is constructed according to the colony−VR complex constructed in the previous step, which is a sequence of simple complexes generated by the increasing proportion parameter σ. According to the distance between all the points recorded in the previous step, specify a value of σ so that each pair of points forms an edge. Therefore, all the simple complexes hidden in each value of σ form a coherent filter stream. Basically, when the longest edge of a simplex complex appears, the domain flow of every simplex in the subcomplex will appear. In order for it to become a filter stream, it needs to have a total order. Total order is the ordering of the simplex in the filter according to the “less than” relation (that is, the “values” of any two simplices are not equal).

The filter value of a simplex depends in part on the length of the longest edge. But sometimes the longest sides of two different simplex forms are the same length, and for any two simplex forms $\tau_{1}$$\tau_{2}$, there are several cases:

1. A 0-dimensional simplex must precede a 1-dimensional simplex, a 1-dimensional simplex must have fewer than 2-dimensional simplices, and so on. This means that any face of a simplex (i.e., f⊂τ) is automatically ordered before the simplex itself. That is:
  (8) $$dim\left(\tau_{1}\right)<dim\left(\tau_{2}\right)\Rightarrow \tau_{1}<\tau_{2}$$
2. If the dimensions of $\tau_{1}$,$\tau_{2}$ are equal, then the value of each simplex is determined by its longest 1-dimensional simplex, that is, its highest gravity. So if $dim\left(\tau_{1}\right)=dim\left(\tau_{2}\right)$, then
  (9) $$max\_edge\left(\tau_{1}\right)<max\_edge\left(\tau_{2}\right)\Rightarrow \tau_{1}<\tau_{2}$$
3. If $\tau_{1}$,$\tau_{2}$ have the same dimension and their longest sides are equal, then the value of each simplex is determined by its largest node. So if $dim\left(\tau_{1}\right)=dim\left(\tau_{2}\right)$ and $max\_edge\left(\tau_{1}\right)=max\_edge\left(\tau_{2}\right)$ at the same time, then
  (10) $$max\_vertex\left(\tau_{1}\right)<max\_vertex\left(\tau_{2}\right)\Rightarrow \tau_{1}<\tau_{2}$$

Thus, the corresponding filtration of the colony complex is obtained, its homology groups are calculated at each filtration step, and the “life cycle” of the topological feature changes through the filtration is tracked. It is through the persistence of these life cycles that persistent coherence reveals the topological properties of the data.

In the process of identifying and constructing the topological structure of the colony, the algorithm not only reveals the geometric shape of the colony growth but also captures its inherent topological properties through detailed analysis and computation of the dataset. This process involves the identification, classification, and ordering of each simplex in the colony to ensure the accuracy and integrity of the topological structure [16]. The output of the algorithm is not merely a series of abstract topological feature values; rather, it maps these features back onto the original image data.

Through this feedback mechanism, the algorithm is able to translate topological insights into visual information, enabling researchers to visually observe the correspondence between the colony’s topology and the original image. This reconstruction not only enhances the interpretability of the dataset but also provides rich contextual information for further analysis of the model. It is easy to combine with subsequent SCoT self-attention mechanisms.

In this process, the algorithm effectively reconstructs the input features of the dataset, transforming the originally complex image data into a series of feature vectors with clear topological significance. As the input of the model, these feature vectors greatly improve the efficiency and accuracy of data processing. More importantly, these deep topological features provide a new classification standard for the model, allowing the model to classify the colony based on its essential structure rather than just surface features.

### 3.4. SCoT_EfficientNet

#### 3.4.1. EfficientNet

EfficientNet is an efficient convolutional neural network architecture proposed by the Google Brain team. It improves the network by uniformly scaling the three dimensions of network depth, network width, and image resolution with a set of fixed scaling coefficients. The number of modules stacked determines the depth of the network, and the width is determined by the number of convolutional kernels in the depth-separable convolution. The size of the input image determines the image resolution. The lightweight inverted bottleneck convolution MBConv is a major component of the EfficientNet model series [34]. The structure of this module is similar to deep separable convolution. First, a 1 × 1 pointwise convolution is performed on the input feature maps to expand feature dimensions, and then deep convolution is used to extract information on high-dimensional features. Finally, the dimension is reduced by 1 × 1 point convolution. In order to focus on key features, the SE (Squeeze and Excitation) channel attention mechanism is introduced after the deep convolution inside the module. The SE attention can give higher weights to channels with large information by weighting information on channel dimensions. All convolution operations in this module are added to batch normalization, and the activation function is Swish. When designing the module, two residual edges are introduced to ensure the flow of deep and shallow information in the module. The MBconv module is designed with a reciprocal residual structure similar to that of the MobileNetV2 network [35] and has a better feature extraction capability. In this paper, the EfficientNet network was structured and improved to further achieve a comprehensive balance of model detection accuracy, model size, and robustness.

#### 3.4.2. SCoT

**Figure 6:**
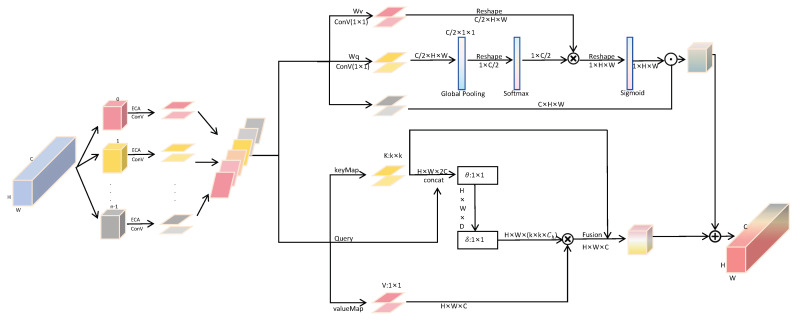
The structure of SCoT attention. After feature segmentation, the approach parallelly considers both spatial and contextual information, with each being weighted differently during the learning process. The results of this learning are then combined and outputted.

The attention mechanism enables the model to focus on relevant information more effectively when processing complex input data, thus improving performance. The attention mechanism can be seen as a dynamic weight allocation that highlights important parts by giving each input element a different weight [36]. Attention mechanisms have been introduced into many visual tasks to address the limitations of standard convolutions [37]. SCoT (Space Contextual Transformer), a new attention mechanism, is introduced in this paper. While capturing spatial correlations in multi-scale input feature maps, it can also be used to understand the temporal dependencies in the sequence data. The spatial information of multi-scale input feature maps can be processed and the long-term dependencies between multi-scale channel attention can be effectively established. The structure of the SCoT attention module is shown in Figure 6.

Firstly, for the two-dimensional effective feature graph *S* of the network input, the size of which is H×W×C (*H*: height, *W*: width, and *C*: number of channels), the feature graph *S* is divided into n parts, represented by $\left[S_{0},S_{1},\ldots ,S_{n-1}\right]$, and the number of channels of each part is $C1=\frac{C}{n}$. Then, the feature graph after each segment is defined as $S_{i}=R^{C1\times W\times H}$, where i=0,1,…,n−1. For each channel feature map, divided, multi-scale convolution kernel grouping convolution is used to extract the spatial information of feature maps of different scales to reduce the number of parameters. The group size is selected according to the size of the convolution Kernel.

Then, we combine spatial channel and contextual information to guide self-attention learning. For the spatial branch part, “polarization filtering” is performed in the attention calculation. The self-attention block operates on the input tensor *X* to highlight or suppress features, much like an optical lens that filters light. Polarization filtering, by allowing only light transmission that is orthogonal to the original direction, can potentially improve the contrast of images. We borrow key elements from photography, folding features completely in one direction while maintaining high resolution in their orthogonal directions. The dynamic range of attention is increased by applying Softmax normalization on the bottleneck tensor (the smallest feature tensor in the attention block), and then tone-mapping is performed using the Sigmoid function. Thus, we obtain $A^{sp}\left(S\right)\in R^{1\times H\times W}$:

(11) $$A^{sp}(S)=F_{SG}[\sigma_{3}(F_{SM}(\sigma_{1}(F_{GP}(W_{q}(S))))\times \sigma_{2}(W_{\upsilon }(S)))]$$

where $W_{q}$ and $W_{v}$ are the standard 1×1 convolution layers; $\sigma_{1}$, $\sigma_{2}$, and $\sigma_{3}$ are the three tensor shaping operators; $F_{SM}$ is the Softmax operator; $F_{GP}$ is a global pool operator; and × is the matrix dot product operation. In this part, the output of the space branch is $Z^{SP}=A^{SP}{⊙}^{SP}$, and ${⊙}^{SP}$ is a multiplication operator on space.

For the branch of combining context information, traditional self-attention can trigger the feature interaction among different spatial locations well, but all pairs of query key relationships are learned independently on isolated query key pairs, without exploring the rich context in the middle. This severely limits the ability of self-attentional learning to learn visual representations on 2D feature maps. To alleviate this problem, this paper builds a unified architecture based on a Transformer that integrates contextual information mining and self-attention learning into a single architecture. Therefore, the context information between adjacent keys is fully utilized to promote self-attention learning in an effective way and enhance the representativeness of the output aggregate feature map. For the feature module *S* consisting of 2D different channels formed by the segmentation in the previous step, its key, query, and value are defined as K=S, Q=S, and $V=SW_{v}$, respectively. Spatially, k×k convolution kernels are used for all adjacent keys in a k×k grid to represent each key in conjunction with the context. The learned context key $K^{1}\in R^{C\times H\times W}$ naturally reflects the static context information between locally adjacent keys and represents the static context of input *S* as $K^{1}$. Then, with the concatenation of static context key $K^{1}$ and query *Q*, the attention matrix is implemented by two successive convolutions of k×k:

(12) $$A=\left[K^{1},Q\right]W_{\theta }W_{\delta }$$

Among them, $W_{\theta }$ has a ReLU activation function and $W_{\delta }$ has no activation function. In this part, the local attention matrix for each spatial position of *A* can be learned based on the query features and the key features of the context, thus enhancing self-attention learning. And according to the context attention matrix *A*, aggregate all values *V* to calculate the participating feature mapping $K^{2}$:

(13) $$K^{2}=V⊗A$$

$K^{2}$ is the dynamic context representation of input *S* and is used to capture dynamic feature interactions among inputs.

Thus, for the branch combining context information, the output $Z^{ct}$ is represented by the attention mechanism as a fusion of static context $K^{1}$ and dynamic context $K^{2}$:

(14) $$Z^{ct}=K^{1}⊗K^{2}$$

The output of the above two branches is composed in a parallel layout:

(15) $$SCoT\left(S\right)=Z^{SP}+Z^{ct}=A^{SP}\left(S\right){⊙}^{SP}\cdot S+K^{1}⊗K^{2}$$

#### 3.4.3. EMBCouv

**Figure 7:**
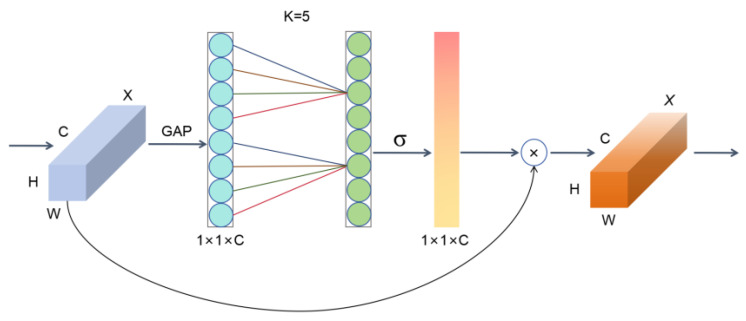
The structure of ECA. The input feature map undergoes a global average pooling operation, followed by convolution with a kernel size of 5 to capture information, which is then processed through an activation function. The result is multiplied with the original input and outputted.

The SE module firstly conducts global average pooling (GAP) on the feature map and then transforms the high-dimensional global feature map into a low-dimensional feature vector through a dimensionality reduction operation to obtain the global feature representation on the channel. However, dimensionality reduction is not conducive to the prediction of the channel attention mechanism, and the extraction ability and efficiency of inter-channel relations are weak [38]. To avoid the negative impact caused by dimensionality reduction in the SE module, the Efficient Channel Attention (ECA) module is used to achieve local cross-channel efficient interaction and reduce the number of parameters. Figure 7 shows the structure of the ECA module.

The ECA module first takes the effective feature graph *X* of the network input, where $X\in R^{H\times W\times C}$, and converts *X* into $X_{1}$, $X_{1}=\in R^{1\times 1\times C}$ through the global average pooling layer. In order to transform the obtained feature graph $X_{1}$ into a shape that meets the needs of subsequent convolution operations, it becomes $X_{3}$, where $X_{3}\in R^{1\times C}$, and the weight of the one-dimensional convolution operation used is

(16) $$w_{i}=\delta \left(\sum_{j=1}^{k}w_{i}^{j}y_{i}^{j}\right),y_{i}^{j}\in \Omega_{i}^{k}$$

where $\Omega_{i}^{k}$ represents the set of *k* adjacent channels of $y_{i}$, and information exchange between channels is realized through a one-dimensional convolution of convolution size *k*:

(17) $$w=\delta (C1D_{k}\left(y\right))$$

where C1D stands for one-dimensional convolution, and the magnitude of *k* is proportional to the channel dimension *C*, and there is a mapping φ between *k* and *C*:

(18) C=φ(k)

If the exponential function with base 2 is used to represent the nonlinear mapping relationship:

(19) $$c=\varphi \left(k\right)=2^{r\times k-b}$$

Finally, the formula for the ECA module to adaptively calculate the convolution kernel size *k* is as follows:

(20) $$k=\varphi \left(c\right)={\left|log_{2}\left(c\right)/r+b/r\right|}_{odd}$$

Then, the output feature map is passed through the sigmoid activation function, and finally, the standardized output is transformed into a dimensional shape $X\in R^{1\times 1\times C}$. Finally, the channel attention weight obtained in the previous step is multiplied by the original input feature map to obtain the final result.

**Figure 8:**
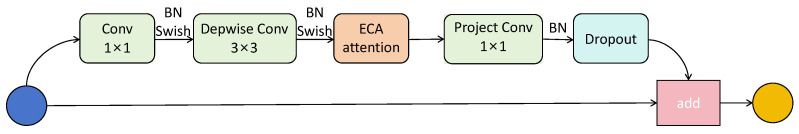
EMBConv structure. The architecture consists of a 1 × 1 standard convolution, a 3 × 3 depthwise convolution, an ECA module, another 1 × 1 standard convolution, and a dropout layer.

The feature extraction network was optimized based on the reconstruction of the ECA module. The original EfficientNet network model used the MBConv module to capture local detailed features in images. Multiple experiments proved that the feature extraction capability of the MBConv module for local small targets was not optimal. In this paper, the ECA module is introduced into the MBConv module and named EMBConv. As shown in Figure 8, the ECA module replaces the SE attention module in the MBConv module with the ECA module. Multi-scale semantic information in the ECA module increases the diversity of colony features and enhances the model’s learning of colony semantic information. It makes the model pay more attention to the details of the colony.

In this paper, the improved EfficientNet feature extraction network was built by combining the improved EMBConv module with the backbone network and adding the SCoT attention module. When colony images are input into the network, the feature information is extracted layer by layer through the convolutional layer and the MBConv module. The extracted features are learned from two aspects of spatial and contextual information through the SCoT attention module, and important and irrelevant features are identified. The network allocates computing resources reasonably according to the importance of the features, thus achieving higher recognition accuracy with fewer parameters.

## 4. Experiment and Results

### 4.1. Experimentation

#### 4.1.1. Experimental Environment and Evaluation Metrics

The experimental environment used in this study is summarized in Table 1:

In this study, the performance of the algorithm was evaluated using the following quantitative metrics: accuracy, precision, recall, F1 score, and the MCC (Matthews Correlation Coefficient) [39]. These metrics are defined mathematically in Equations (21)–(25):

(21) $$Accuracy=\frac{TP+TN}{TP+TN+FP+FN}$$

(22) $$Precision=\frac{TP}{TP+FP}$$

(23) $$Recall=\frac{TP}{TP+FN}$$

(24) $$F-score=\frac{2TP}{2TP+FP+FN}$$

(25) $$MCC=\frac{TP\times TN-FP\times FN}{\sqrt{\left(TP+FP)(TP+FN)(TN+FP)(TN+FN\right)}}$$

Accuracy reflects the ratio between the number of samples correctly classified by the classifier and the total number of samples; precision measures the proportion of true positives among the samples predicted as positive by the classifier; and recall represents the ratio of true positives correctly predicted as positive by the classifier to all true positives. The F1 score is the harmonic mean of precision and recall, which is used to comprehensively evaluate the classifier’s performance. The MCC is a comprehensive performance metric for binary classification problems, considering the balance of true positives, false positives, true negatives, and false negatives to assess the overall effectiveness of the classifier. A comprehensive analysis of these performance metrics provides a quantitative basis for evaluating the classifier’s classification capabilities across different categories and assists decision makers in selecting the most suitable model or adjusting model thresholds when facing specific tasks.

#### 4.1.2. Persistent Homology

**Figure 9:**
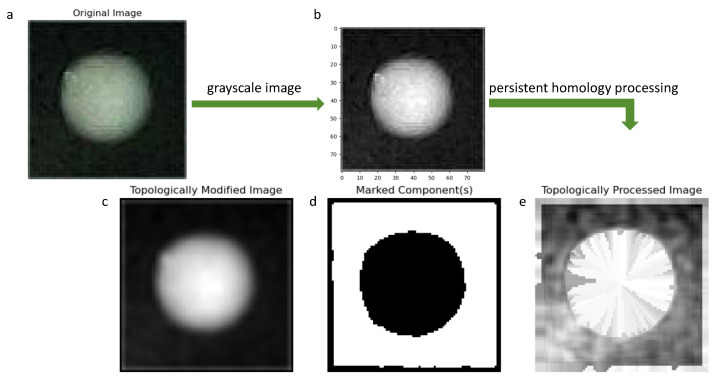
PH treatment result diagram. Figure (**a**) represents the original image of the dataset without PH feature extraction, Figure (**b**) represents the gray image after gray level processing of the original image, and (**c**) is the topological gray level image filtered by PH filter. Here, the window_size parameter is generally set to 5. The border (border_width) parameter is set to 1 to process the image. (**d**) image is the post-test editing component after PH processing, and (**e**) image is the final image data to extract topological image features.

The effective features of colony images in the dataset are limited, and the features are not obvious or prominent. Therefore, the PH feature extraction method is introduced. The topological features of the PH detected dataset were introduced into the collected colony images to form a new feature dataset including the extracted topological information, as shown in Figure 9.

**Figure 10:**
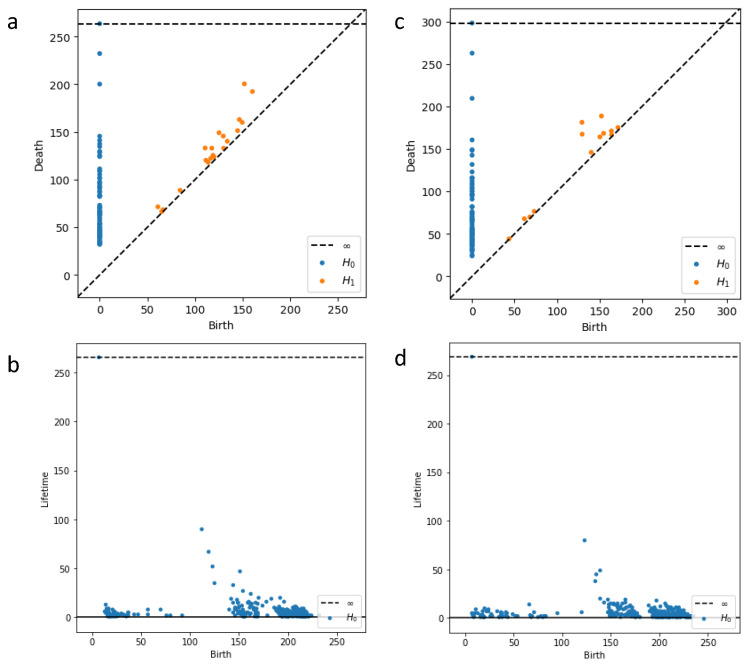
PD representation of CA and SE. Figures (**a**,**b**) represent the PD representation and PD variant of the CA dataset, while figures (**c**,**d**) represent the PD representation and PD variant of the SE dataset. $H_{0}$ denotes the persistence of the component in 0 dimensions, and $H_{1}$ signifies the persistence of the component in 1 dimension, which is the persistence of gaps between data points.

The Persistence Diagram (PD) generated by PH can be used as a feature representation of the data. These features capture the topological properties of the dataset and have scale invariance to a certain extent. It can be clearly seen from Figure 10 that after VR complex construction of PH and filtering, different topological components of the data are represented by birth time and death time, and their life cycle is calculated. Components whose life cycle is greater than the threshold value are selected, and any components other than the selected components are eliminated, thus obtaining the final topological image.

**Figure 11:**
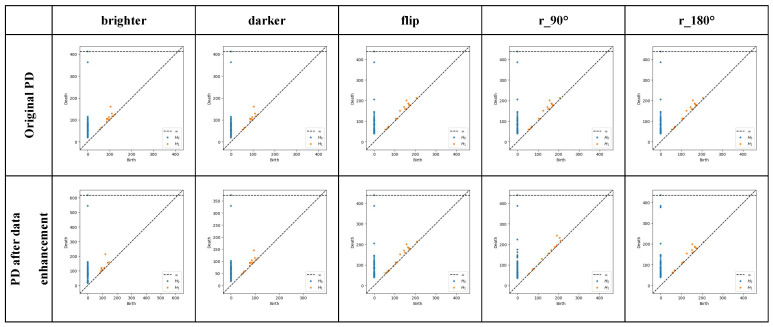
PD comparison graph under five data enhancement methods. The selected five data augmentation methods do not significantly affect the results of PH processing.

Figure 11 shows the original image at the top and the images augmented by the data enhancement method corresponding to the original image at the bottom. As can be seen from Figure 11, the five data enhancement methods used in image preprocessing, brighter, darker, flip, 90° to the right rotation, and 180° rotation [40], can retain the extracted topological components and do not affect the feature extraction results while expanding the dataset.

To further validate the effectiveness of PH in bacterial colony processing, this paper integrates the PH algorithm with classical classification models, including EfficientNet, MobileNet, ResNet [41], and ResNeXt [42], for comparative experiments. The experimental results are presented in Table 2.

Experiments show that PH and various classification models have achieved a good combined performance. By comparing the results, it can be seen that the combination effect of the method used in this experiment is the best, and the comprehensive value basically reaches the best level. PH combined with the MobileNet network has the best recall rate, reaching 97.95%, but the other four evaluation indicators are low and the performance is not stable. Besides the methods used in this experiment, the combination of PH and EfficientNet is the most robust, which also proves the necessity and superiority of the combination of the two methods. In general, the PH-treated colony dataset fed into the model classification training improves all kinds of deep learning classification models to varying degrees. This also means that on small-scale and inconspicuous colony datasets, the use of PH for feature extraction can reliably detect and label the invisible features of the dataset, ensuring the reliability of subsequent identification and classification and improving the accuracy of research results.

#### 4.1.3. SCoT_EfficientNet

After the PH feature processing, the dataset needs to be fed into the classification model. Here, this paper selects EfficientNet as the classification model. It optimizes the network structure by combining the scaling methods of the depth, width, and resolution of the network, and its advantage is that the efficiency and accuracy of the model are optimized and balanced, so as to achieve a better performance in the case of limited computing resources. Compared to the SE attention mechanism used by the MBConv structure in traditional EfficientNet, the ECA mechanism can better apply an adaptive volume kernel to each channel to calculate the channel attention weight, thus achieving the perception of different channel features rather than reducing the dimension of channel features to scalars. Following model experimentation, the resulting EMBConv structure replaces the second stage section of the original EfficientNet to better fit the new dataset of topological features extracted via PH.

EMBConv interacts with MBConv to learn channel information in both local and global dimensions to ensure the diversification of feature extraction. However, the feature weights in the spatial dimension of the model are ignored to some extent. The components constituting the topological features of the image are interrelated, reflecting the multilevel nature, stability, and persistence of the topological structure. Therefore, in this paper, a new self-attention mechanism called SCoT is created, which is weighted in the dual dimensions of space and scene conditions, so that the model can not only enhance the perception ability of the model by using the relationship between pixels in the local area but also adjust the distribution of attention according to different context information and conditions, so as to deal with complex scenes and multi-object situations.

Upon model validation, this study connects the SCoT self-attention mechanism after the eighth layer stage. The data processed by the EMBConv and MBConv structures are then input into SCoT for weight redistribution, reorganization of computational resources, and subsequent rational classification through convolutional layers, thereby enhancing classification accuracy and computational efficiency.

In order to verify the necessity and accuracy of the improved model, ablation experiments were designed for the improved SCoT_EfficientNet method. Separate experiments were performed on EMBConv structure improvement and SCoT improvement, and the original EfficientNet models without improvement were compared. The dataset after PH treatment was input into four model experiments, and the results are shown in Table 3.

It can be clearly seen from Table 3 that after the introduction of PH treatment, the classification results of the EfficientNet models all performed well, and the training results gradually improved with the improvement in the models. Among them, the improved method used in this paper shows the best comparison results in the five dimensions. This also reflects the advanced nature and necessity of the improvement in the SCoT_EfficientNet model.

### 4.2. Experimental Outcomes

**Figure 12:**
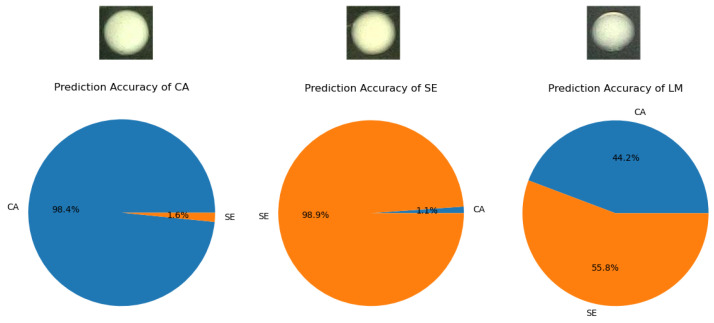
Model classification accuracy graph. In this study, the model achieved a classification accuracy of 98.4% for CA and 98.9% for SE, but was unable to accurately distinguish the novel colony LM.

The dataset is tested through the above steps. As shown in Figure 12, during the testing process, the accuracy for CA colony classification and SE colony classification for CA and SE colony classification can reach 98.4% and 98.9%, respectively. In order to ensure the rigor and reliability of the experiment, a third colony LM (Listeria Moncytogenes) was introduced in this paper [43]. LM has certain similarities with CA and SE in appearance, making it difficult to distinguish visually. The LM dataset processed under the same culturing conditions was then imported into the training model. As can be seen from Figure 12, the model could effectively discriminate against the interference term LM, indicating that the experimental model had a high degree of accuracy and specificity.

The overall ablation experiment results are shown in Table 4. Cross-ablation experiments were performed on the SCoT_EfficientNet model combined with the PH treatment, the SCoT_EfficientNet model without the PH treatment, the PH-treated EfficientNet model, and the single EfficientNet model, followed by a detailed analysis of the experimental results. Overall, the experimental method adopted in this study achieved improvements to varying degrees across the five evaluation dimensions, and the results were superior to those of the other three models. In addition, the SCoT_EfficientNet model without the PH treatment and the PH-treated EfficientNet model showed better results than the untreated ones. Although the EfficientNet model retained an excellent precision score of 97.62%, the other four evaluation indicators were lower than the training scores of the other three models, and the precision value was also lower than the 98.89% used in the experiment.

In order to validate the superiority of the proposed model, classical classification models such as GoogleNet [44], MobileNet, ResNet, ResNeXt, and ViT [45] were introduced to conduct classification training on the experimental training dataset, and the five classification indices discussed above were introduced again for evaluation. The results are shown in Table 5. As can be seen from Table 5, the method adopted in this study achieved varying degrees of improvement compared with the above classical algorithms across the five evaluation metrics. Although MobileNet and EfficientNet scored 91.30% and 97.62% in precision, ResNeXt scored 92.31% in recall, and the results were not as good as those obtained by experimental methods. It is worth noting that the performance of ViT on this dataset was not satisfactory, which also indicates that ViT is not a good choice for small-scale datasets, and EfficientNet is a more suitable alternative to consider. Additionally, MSAs can lead to negative Hessian eigenvalues in small data regimes.

To comprehensively evaluate the practicality of the proposed method, additional metrics including Params, FLOPs, and inference time were introduced to assess the model’s performance from multiple perspectives. Experimental comparisons with classical models were also conducted to analyze computational costs. The detailed experimental results are presented in Table 6.

According to the data presented in the comparison table, our proposed method exhibits a slight increase in the number of parameters and FLOPs compared to the EfficientNet baseline, yet it still outperforms most classical models. Additionally, our method achieves an average single-image inference time of approximately 0.022 s. These results indicate that although our approach introduces a certain degree of theoretical computational complexity, in practical applications, the additional computational cost remains moderate and reasonable, without significantly impacting the model’s usability. On the contrary, this modest increase in computational cost yields substantial performance improvements, clearly demonstrating the efficiency and practical value of our proposed method.

In summary, the colony binary classification method based on Persistent Homology feature extraction technology and the improved EfficientNet effectively learns the topological features of the data and demonstrates good adaptability in practical applications. This research exhibits excellent stability in performing classification tasks, effectively distinguishing between CA and SE bacterial data, and achieving a high accuracy rate.

## 5. Discussion

Future research will focus on the following aspects:

1. Expanding the scope of application. The current experimental model can only classify CA and SE colonies with normal morphology. In the future, we plan to extend its application to colonies with overlapping structures, greater noise interference, and other colony types, thereby completing classification tasks involving multiple colony categories, diverse morphologies, and various colony forms. Furthermore, we will explore the robustness of our methods under varying environmental conditions and colony densities, thereby improving the generalizability and reliability of the classification model.
2. Incorporation of object detection algorithms [46]. Current experiments have been conducted exclusively on isolated bacterial colonies. To better meet practical application requirements, future studies will incorporate object detection algorithms to reduce the preprocessing complexity. This will facilitate the accurate identification and enumeration of bacterial colonies in scenarios where multiple bacterial species coexist. Specifically, we aim to evaluate state-of-the-art deep-learning-based object detection frameworks to determine the most suitable approach for our application. Additionally, we will investigate the integration of object detection and classification tasks into a unified pipeline, potentially enhancing the efficiency and accuracy of colony analysis.
3. Workflow integration. To ensure the practical implementation of the proposed methods, future work will aim to integrate these approaches into clinical workflows. Specifically, we plan to develop a user-friendly visualization interface and establish a comprehensive, streamlined operational protocol. By deploying these tools within commonly used clinical systems, we seek to effectively address the challenge of early-stage classification between CA and SE colonies. Furthermore, we will collaborate closely with clinical microbiologists and laboratory technicians to ensure the developed system aligns with actual clinical needs and laboratory practices. User feedback will be systematically collected and analyzed to iteratively refine the interface design and workflow integration, ultimately facilitating the acceptance and widespread adoption of our proposed methodology in clinical settings.

## 6. Conclusions

This study proposes a binary classification method for colonies based on Persistent Homology feature extraction technology and an improved EfficientNet. By constructing a VR complex using PH and extracting effective topological features of colony data with filters, irrelevant variable information is removed. Subsequently, the processed dataset is input into an improved classification model, SCoT_EfficientNet, which incorporates the ECA module in the EMBConv structure and the SCoT self-attention mechanism with context and spatial attention for classification training. This allows for the sensitive recognition of features extracted by PH, which is more conducive to small target processing, while maintaining efficiency and the lightweight nature of computational efficiency to obtain the final classification results. The accuracy, precision, recall, F-score, and MCC of this method reach 98.64%, 98.89%, 98.42%, 98.65%, and 97.29%, respectively. Therefore, this method can provide efficient and reliable guidance for the classification of CA and SE in real-world scenarios. Ultimately, these research findings can deliver practical benefits to medical professionals, demonstrating significant theoretical value and extensive application potential.

## Figures and Tables

**Table 1:** Experimental environment.

Component	Specification
Operating System	Ubuntu 22.04.5 LTS (GNU/Linux 6.8.0-57-generic x86_64)
CPU	Intel(R) Xeon(R) W-2295 CPU @ 3.00 GHz
RAM	128 GB
GPU	NVIDIA RTX A6000 (48 GB)
GPU Driver Version	575.51.03
CUDA Version	12.9
Storage	SSD: 1 TB; HDD: 4 TB

**Table 2:** Comparative experiments of PH combined with various classification networks.

Networks	Accuracy	Precision	Recall	F-Score	MCC
PH + EfficientNet	0.9505	0.9468	0.9558	0.9513	0.9010
PH + MobileNet	0.8778	0.8158	0.9795	0.8902	0.7711
PH + ResNet	0.9535	0.9286	0.9447	0.9366	0.8707
PH + ResNeXt	0.9353	0.9272	0.9463	0.9367	0.8707
PH + SCoT_EfficientNet	0.9864	0.9889	0.9842	0.9865	0.9729

**Table 3:** SCoT_EfficientNet ablation experiment.

PH_Networks	Accuracy	Precision	Recall	F-Score	MCC
EfficientNet	0.9505	0.9468	0.9558	0.9513	0.9010
ECA + EfficientNet	0.9784	0.9840	0.9731	0.9786	0.9869
SCoT + EfficientNet	0.9760	0.9748	0.9779	0.9763	0.9521
ECA + SCoT + EfficientNet	0.9864	0.9889	0.9842	0.9865	0.9729

**Table 4:** Overall model ablation experiment results table.

Networks	Accuracy	Precision	Recall	F-Score	MCC
EfficientNet	0.8835	0.9762	0.7885	0.8723	0.7822
PH + EfficientNet	0.9505	0.9468	0.9558	0.9513	0.9010
SCoT_EfficientNet	0.9515	0.9796	0.9231	0.9505	0.9045
Our Method	0.9864	0.9889	0.9842	0.9865	0.9729

**Table 5:** Comparative experiment.

Networks	Accuracy	Precision	Recall	F-Score	MCC
GoogleNet	0.835	0.8889	0.7692	0.8247	0.6766
MobileNet	0.8641	0.9130	0.8077	0.8571	0.7334
ResNet	0.835	0.7612	0.9808	0.8571	0.6994
ResNeXt	0.8349	0.7869	0.9231	0.8496	0.6798
EfficientNet	0.8835	0.9762	0.7885	0.8723	0.7822
ViT	0.8058	0.82	0.7885	0.8039	0.6122
Our method	0.9864	0.9889	0.9842	0.9865	0.9729

**Table 6:** Computational costs and efficiency comparison experiments.

Networks	Params (M)	FLOPs (G)	Inference Time (s)
GoogleNet	6.8	1.5	0.019
ResNet	25.5	4.1	0.009
ResNeXt	25	4.3	0.025
ViT	86	17.6	0.041
EfficientNet	5.3	0.39	0.028
Our Method	5.8	0.52	0.022

## Data Availability

Data will be made available upon request.
